# How does digital government influence public policy compliance in public health emergencies? —A study based on mixed method

**DOI:** 10.3389/fpsyg.2025.1518585

**Published:** 2025-05-09

**Authors:** Rongmian Huo, Feimin Liu, Li Wu

**Affiliations:** ^1^School of Humanities and Foreign Languages, China Jiliang University, Hangzhou, China; ^2^Faculty of Arts and Sciences, Beijing Normal University, Zhuhai, China

**Keywords:** policy compliance, digital government, administrative burden, violation costs, digital literacy

## Abstract

The evolution of digital government is reshaping the dynamics of government-citizen interactions, fostering new modes of engagement, co-creation, and participatory governance. This study explores the impact of digital government, administrative burdens, and violation costs on public policy compliance, with a focus on public health emergencies. In Study 1, a survey of 697 participants from regions with different levels of digital government (high, medium, and low) was conducted. The findings indicate that perceptions of the usefulness, ease of use, and transparency of digital government systems are significant predictors of policy compliance, mediated by the administrative burden experienced by citizens. Additionally, digital literacy was found to moderate the relationship between administrative burden and compliance, highlighting the role of digital skills in public policy compliance. In Study 2, an experimental survey with 312 participants examined how violation costs influence the impact of digital government on policy compliance. Results show that violation costs significantly moderate this relationship, aligning with the theoretical framework of loss aversion. The findings offer insights into the boundary conditions under which digital government initiatives can effectively enhance policy compliance in the context of public health emergency, contributing to the broader discourse on governance and public policy implementation in digital contexts.

## 1 Introduction

With the rapid advancement of technologies such as big data, artificial intelligence, and blockchain, governance frameworks including their concepts, rules, systems, and practices are undergoing continuous transformation. Digitalization has emerged as a strategic imperative for modernizing national governance, enhancing both institutional frameworks and governance capacity. Building upon the foundations of e-government, digital government signifies a comprehensive digital transformation of the public sector, aiming to establish a new governance paradigm (Ma, 2021). Innovative approaches such as “big data + grid management,” “government-service mini-programs,” and “health codes” have reshaped administrative interactions, greatly enhancing efficiency, accommodating diverse public demands, and advancing societal welfare (Chen and Jiang, 2024; Gao and Li, 2023). They also play a pivotal role in transforming risk governance strategies and fostering community resilience (Men et al., 2023). Furthermore, public policy compliance is integral to effective policy implementation, as it reflects the degree of public acceptance of collective decisions made by governing bodies. Digital government initiatives are increasingly recognized as critical drivers for enhancing public adherence to policies, promoting seamless governance, and strengthening citizen-state relations (Wen and Li, 2023).

Despite these benefits, rapid digital government expansion has exposed disparities in government–citizen interactions across regions and demographic groups (Zeng and Chen, 2024; Wang and Yi, 2022). Digitally disadvantaged populations often face social exclusion at constructive, instrumental, and passive levels, amplifying governance inequalities among different socioeconomic strata (Jiang and Tang, 2023). Scholars have emphasized that the design and functionality of digital platforms are critical for effective governance, with public trust emerging as a central determinant of platform success (Wang et al., 2023; Jing and Liu, 2023). Furthermore, the processes of government–citizen interaction matter substantially; timely and responsive digital government services strongly affect public satisfaction (Wang and Yi, 2022). While some researchers maintain that digital government can promote broader public participation by leveraging digital capital and enhancing transparency (Xu and Tang, 2020; Wu et al., 2020), others caution that digital governance may yield deeper and more pervasive negative consequences (MacLean and Titah, 2022; Cao and Ma, 2023). Further studies should explore the conditions under which digital government effectively promotes compliance, with particular attention to trust-building mechanisms, the role of public satisfaction, and the effects of responsive governance.

In light of the sudden onset and urgent nature of public health emergency, the present study concentrates on government–citizen interactions during the COVID-19 pandemic to investigate how digital government influences policy compliance. Drawing upon both questionnaire surveys and behavioral experiments, the research examines the mediating and moderating pathways that shape compliance behaviors. Anchored in the theoretical framework of behavioral public administration, this study aims to construct an integrative model that clarifies the conditional mechanisms by which digital government initiatives strengthen public compliance to policies, thereby contributing to a deeper understanding of the evolving relationship between citizens and administrative authorities in the digital era.

## 2 Theoretical review and research hypotheses

### 2.1 Digital government and public policy compliance: the perspective of administrative burden

Digital government leverages internet and information technology to create interactive platforms that transform how administrative authorities engage with citizens. This transformation reorients governance from a “closed” to an “open” operational style and from a “power-oriented” to a “service-oriented” model, thereby enhancing governance efficiency (Wei et al., 2023). To understand how digital government influences public policy compliance, it is useful to draw on the concept of administrative burden, which encompasses the learning, psychological, and compliance costs citizens incur when interacting with government agencies (Moynihan et al., 2015; Ma, 2022). On the one hand, digital government can reduce administrative burden by automating social welfare services, lowering informational barriers, and promoting procedural justice and fair treatment (Fox et al., 2020; Miller and Keiser, 2021; Xu and Tang, 2020). On the other hand, automation and digitally mediated interactions may convert two-way “ask-and-answer” exchanges into one-way human–computer dialogues, thereby requiring higher levels of digital literacy and potentially introducing new administrative burdens (Larsson, 2021; Linos et al., 2022; Cao and Ma, 2023).

The Technology Acceptance Model (TAM) highlights the significance of perceived usefulness and perceived ease of use in shaping public acceptance of digital government. In parallel, research on transparent governance underscores the essential role of information transparency in the development and adoption of digital government initiatives (Venkatesh and Davis, 2000; Wu et al., 2020; Li et al., 2023). These dimensions—ease of use, usefulness, and transparency—collectively influence the public’s perceptions and engagement with digital government platforms, laying the foundation for trust and effective interaction.

Perceived usefulness refers to the extent to which individuals believe that using digital government services will enhance their ability to complete tasks efficiently. It reflects the public’s overall assessment of the functionality and effectiveness of digital government. Functionally, digital government offers interactive platforms that increase accessibility, ease psychological burdens, and lower compliance costs. Additionally, positive prior experiences with government interactions shape higher expectations for the efficiency of digital services, fostering greater public policy compliance.

*H1*: The perceived usefulness of digital government has a significant positive effect on public policy compliance, with administrative burden functioning as a mediating variable.

Perceived ease of use refers to the level of effort the public perceives as necessary to effectively engage with digital government services. It captures the public’s assessment of the usability and accessibility of these platforms, focusing on the cognitive and operational costs associated with their use. High perceived ease of use can significantly reduce learning and compliance costs, thereby alleviating the overall administrative burden and facilitating smoother government-citizen interactions.

*H2*: The perceived ease of use of digital government has a significant positive effect on public policy compliance, with administrative burden serving as a mediating variable.

Information transparency narrows informational asymmetries between administrative authorities and citizens, safeguards the public’s right to know, and fosters openness, all of which can mitigate the psychological burdens frequently associated with government–public interactions. Li et al. (2023) introduced the concept of public-friendly information disclosure, emphasizing the importance of providing accessible and comprehensible information to enhance public engagement and trust.

*H3*: Perceived information transparency in digital government has a significant positive effect on public policy compliance, with administrative burden functioning as a mediating variable.

### 2.2 Administrative burden and public policy compliance: the moderating role of digital literacy

Digital government streamlines and standardizes administrative tasks, enhancing efficiency. However, this standardization may not adequately address the diverse needs, personal circumstances, and varying levels of human capital across different population groups, potentially exacerbating the digital divide. Automated decision-making processes often restrict operational discretion, heightening learning and psychological costs in digital government contexts (Peeters, 2023).

Digital literacy, which denotes an individual’s capacity to access information and navigate digital platforms, is a critical personal resource influencing how citizens handle these costs (Döring, 2021). From a resource-based perspective, citizens with high digital literacy generally manage administrative burdens more effectively and thereby maintain higher compliance (Zhang and Yang, 2023; Wu and Wang, 2023). In contrast, those with low digital literacy often struggle, underscoring that the efficacy of digital government varies considerably across population segments. These disparities highlight that the impact of digital government varies across groups, positioning digital literacy as a key moderating factor in determining the extent to which individuals can engage with and benefit from digital public services.

*H4*: Administrative burden has a negative effect on policy compliance, with public digital literacy moderating the strength of this relationship.

### 2.3 Administrative burden and violation costs: a conditional process model

To capture the complexity of how costs and benefits shape compliance, a conditional process model is instructive. From a policy-evaluation standpoint, public adherence hinges on reducing the cost of compliance or heightening the cost of noncompliance (Barnes and Petry, 2021; Li et al., 2021). Digital government initiatives enhance information transparency and standardize administrative processes, thereby lowering compliance costs for the public. However, the impact of digital government on policy compliance depends on how individuals respond to varying policy scenarios, particularly when noncompliance costs differ. Understanding the conditional effects of digital government across different noncompliance cost contexts is essential for designing effective policy interventions.

**Figure 1:**
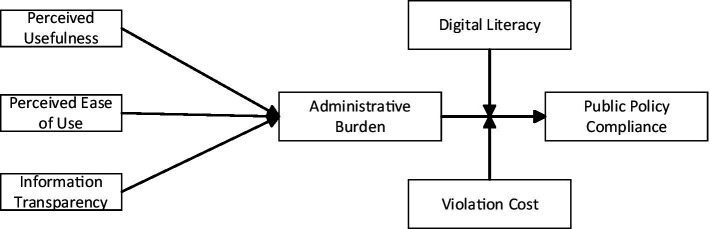
Theoretical model of the study.

Loss-aversion theory (Kahneman et al., 1991) indicates that individuals are more motivated to avoid negative outcomes than to pursue equivalent positive ones, a phenomenon recently extended to information contexts (Litovsky et al., 2022). When violation costs are high, citizens show a greater propensity to accept administrative burdens in order to avoid penalties, thereby mitigating the negative impact of such burdens on compliance. When violation costs are low, however, that willingness declines, causing administrative burdens to weigh more heavily on compliance decisions. These discussions emphasize the complex interplay between administrative burdens and compliance incentives, offering critical insights on how governments can enhance policy. Hypothesis 5 captures this idea by proposing that violation costs moderate the effect of administrative burden on policy compliance. Figure 1 illustrates the overarching conceptual framework that connects digital government, administrative burden, public digital literacy, and violation costs in shaping public policy compliance behavior.

*H5*: The effect of administrative burden on policy compliance is moderated by violation costs.

*H5a*: When violation costs are low, administrative burden negatively impacts policy compliance.

*H5b*: When violation costs are high, the negative impact of administrative burden on policy compliance is diminished.

## 3 Study 1: the impact of digital government on public policy compliance in public health emergencies: the mediating role of administrative burden

### 3.1 Participants

This study utilized the Credamo platform for online questionnaire distribution. Drawing on the Digital China Development Report (2020) issued by the National Internet Information Office to select two administrative regions from each of three tiers distinguished by varying levels of digitalization: high, medium, and low. Specifically, Shanghai Municipality and Zhejiang Province represented the high-digitalization tier, Jiangxi Province and Guizhou Province comprised the medium tier, and Inner Mongolia Autonomous Region and Xinjiang Uygur Autonomous Region were categorized as the low tier. Of the 697 completed questionnaires, 58 were excluded due to failing attention-check items, resulting in 639 valid responses (91.67% valid-response rate). Responses were evenly distributed across tiers, with 215 from first-tier regions and 212 each from the second- and third-tier regions, ensuring balanced representation of different digitalization levels.

Among the 639 valid participants, 234 (36.6%) identified as male and 405 (63.4%) as female. The age distribution was diverse: 3 participants (0.5%) were under 18, 231 (36.2%) were aged 18–25, 151 (23.6%) were 26–35, 79 (12.4%) were 36–45, and 175 (27.4%) were 46 or older. With respect to educational background, 25 participants (3.9%) had completed junior high school or below, 67 (10.5%) had finished high school, and 547 (85.6%) had attained a university-level education or higher. Data collection occurred in December 2022, before the full reopening following the COVID-19 pandemic.

### 3.2 Measures

Perceived Usefulness was measured using the scale developed by Venkatesh and Davis (2000). Participants rated each item on a 5-point Likert scale (1 = strongly disagree to 5 = strongly agree). The scale included four items, such as: ‘Using the digital system for epidemic prevention increases my efficiency in controlling the epidemic’ and ‘Using the digital system for epidemic prevention improves the quality of my epidemic control.’ *α* = 0.794.

Perceived ease of use was measured using the validated scale developed by Venkatesh and Davis (2000). Participants responded to four items on a 5-point Likert scale (1 = strongly disagree to 5 = strongly agree). Sample items included: ‘Interacting with the digital system for epidemic prevention is easy for me to understand’ and ‘Overall, I find the digital system for epidemic prevention easy to use’ *α* = 0.807.

Information transparency was measured using the scale developed by Wu and Ma (2020). Participants evaluated seven items on a 5-point Likert scale (1 = strongly disagree to 5 = strongly agree). Sample items included: ‘I believe the central government utilizes the digital system for epidemic prevention to release and update epidemic information promptly’ and ‘The city government where I reside uses the digital system for epidemic prevention to release and update epidemic information promptly.’ *α* = 0.838.

Administrative burden was measured using a scale developed from the works of Döring and Madsen (2022) and Madsen and Mikkelsen (2022). The scale included five items, assessing three dimensions of burden: learning costs, psychological costs, and compliance costs. An example item for learning costs is, ‘I often spend time learning how to operate the digital system for epidemic prevention.’ For psychological costs, an item reads, I often feel anxious about struggling to master the use of the digital system for epidemic prevention. An example of compliance costs is, Under the management of the digital system for epidemic prevention, I have to meet many requirements (such as entry and exit inspections, path tracking, etc.), which restricts me from arranging my daily life freely.

Digital literacy refers to the ability to effectively engage with diverse data sources and digital platforms (Döring, 2021). This study employed the CSS2019 scale, comprising six items. Sample items include: ‘I can use a computer to access government websites’ and ‘I can use a smartphone to download and install digital government apps’ α = 0.776.

Public policy compliance with COVID-19 regulations was assessed using a three-item scale. Sample items include: ‘The transparency and convenience of the digital reform process encourage me to follow regulations and get vaccinated against COVID-19,’ ‘The convenience of the process motivates me to comply with local COVID-19 testing requirements,’ and ‘Overall, I adhere to COVID-19 prevention regulations under the framework of digital governance’ α = 0.761.

### 3.3 Results

#### 3.3.1 Common method bias test and correlation analysis

A Harman’s single-factor test was conducted to evaluate the potential impact of common method bias (Podsakoff et al., 2003). The analysis revealed that the first factor accounted for 32.54% of the total variance, remaining below the commonly accepted 40% threshold. Consequently, common method variance does not appear to pose a significant threat to the validity of this study’s findings. Descriptive statistics and Pearson correlation coefficients for the variables are presented in Table 1.

**Table 1:** Descriptive statistics and correlation coefficients.

Variable	*M*	*SD*	1	2	3	4	5	6
1. Perceived Usefulness	4.19	0.65	1					
2. Perceived Ease of Use	4.09	0.69	0.588***	1				
3. Information Transparency	4.04	0.65	0.666***	0.595***	1			
4. Digital Literacy	4.33	0.56	0.511***	0.435***	0.530***	1		
5. Administrative Burden	2.51	0.92	−0.308***	−0.328***	−0.342***	−0.285***	1	
6. Public Policy Compliance	4.47	0.57	0.484***	0.447***	−0.511**	0.513***	−0.406***	1

**p < 0.01, ***p < 0.001.

#### 3.3.2 A hierarchical analysis of administrative burden’s impact on public policy compliance across different levels of digital government

Drawing on the Digital China Development Report (2020), Shanghai Municipality and Zhejiang Province represented the high-digitalization, Jiangxi Province and Guizhou Province represented the medium, and Inner Mongolia Autonomous Region and Xinjiang Uygur Autonomous Region were categorized as the low. To examine the differences in administrative burden and public policy compliance among cities with varying levels of digital government, we conducted a homogeneity-of-variance test, which showed that neither administrative burden nor public policy compliance met the homogeneity assumption (*p* < 0.001). Consequently, Welch’s test was employed to evaluate the group-based differences, as presented in Table 2. The results show significant differences in the mean values of administrative burden (Welch’s *F* = 42.828, *p* < 0.001) and public policy compliance (Welch’s *F* = 16.404, *p* < 0.001) across the three groups, suggesting a potential link between higher administrative burden and lower compliance.

**Table 2:** ANOVA analysis of administrative burden and public policy compliance under different levels of digital government.

Variable	Digital government	*N*	*M*	*SD*	*SE*	95% CI Lower	95% CI Upper	Welch
Administrative burden	High	215	2.30	0.79	0.05	2.19	2.40	
	Media	212	2.23	0.71	0.05	2.13	2.32	42.82^***^
	Low	212	2.99	1.03	0.07	2.85	3.13	
	Total	639	2.51	0.92	0.04	2.43	2.58	
Public policy compliance	High	215	4.44	0.60	0.04	4.36	4.52	
	Media	212	4.62	0.41	0.03	4.57	4.68	16.40^***^
	Low	212	4.34	0.64	0.04	4.26	4.43	
	Total	639	4.47	0.57	0.02	4.42	4.51	

***p < 0.001.

A further analysis was conducted to examine the impact of administrative burden on public policy compliance under varying levels of digital government. Using SPSS 26.0 with the PROCESS (Model 1), we tested whether the level of digital development moderates the relationship between administrative burden and public policy compliance. The results are presented in Table 3.

**Table 3:** The moderating effect of digital government.

Variable	*B*	*SE*	*t*	*p*	95% CI Lower	95% CI Upper
Constant (Intercept)	5.1092	0.1986	25.7247***	<0.001	4.7192	5.4992
Age	−0.01	0.0182	−0.5478	0.584	−0.0458	0.0258
Edu	−0.0155	0.0456	−0.3393	0.7345	−0.1051	0.0741
Adbur (X)	−0.2576	0.0449	−5.7428***	<0.001	−0.3457	−0.1695
DG = 2 vs. 1 (W1)	0.067	0.1595	0.4201	0.6746	−0.2462	0.3802
DG = 3 vs. 1 (W2)	−0.0015	0.1651	−0.0093	0.9926	−0.3257	0.3227
Adbur × W1 (Int_1)	0.0431	0.0671	0.6422	0.521	−0.0887	0.1749
Adbur × W2 (Int_2)	−0.0376	0.0624	−0.6015	0.5477	−0.1601	0.085

***p < 0.001.

The result indicates that administrative burden exerts a significant negative effect on public policy compliance. The interaction between digital government level and administrative burden is not significant, suggesting that in this study’s sample, differences among levels of digital government do not substantially alter how administrative burden influences policy compliance.

#### 3.3.3 The mediating effect of administrative burden

Controlling for educational level and age, the results demonstrate that perceived usefulness positively predicts public policy compliance (*β* = 0.498, *p* < 0.001), with an adjusted *R^2^* = 0.244 and *ΔR^2^* = 0.244. The overall model was statistically significant (*F* (1, 635) = 205.96, *p* < 0.001). Similarly, perceived ease of use positively predicts public policy compliance (*β* = 0.456, *p* < 0.001), yielding an adjusted *R^2^* = 0.244 and *ΔR^2^* = 0.205, with the model being significant (*F* (1, 635) = 165.169, *p* < 0.001). In addition, information transparency is a significant positive predictor of public policy compliance (*β* = 0.528, *p* < 0.001). The model achieved an adjusted *R^2^* = 0.274 and *ΔR^2^* = 0.274, with statistical significance (*F* (1, 635) = 240.771, *p* < 0.001). Conversely, administrative burden negatively predicts public policy compliance (*β* = −0.404, *p* < 0.001), with an adjusted *R^2^* = 0.161 and *ΔR^2^* = 0.162. The model was statistically significant (*F* (1, 635) = 123.201, *p* < 0.001).

To further examine whether administrative burden mediates the effect of these independent variables on policy compliance, the PROCESS plugin for SPSS 26.0 (Model 4) was employed, following Hayes’s (2018) recommendations. In this mediation analysis, perceived usefulness, perceived ease of use, and information transparency served as predictors, administrative burden was the mediator, and public policy compliance was the outcome variable; educational level and age were included as control variables. The path coefficients are summarized in Table 4.

**Table 4:** Mediating effects of administrative burden.

Predictor variable	Administrative burden (DV: M)			Policy compliance (DV: Y)		
	*β*	*SE*	*t*	*β*	*SE*	*t*
Age	0.12	0.03	2.94**	−0.08	0.02	−2.48
Educational Level	0.05	0.08	1.21	−0.04	0.04	−1.08*
X1: Perceived Usefulness	−0.32	0.05	−8.49***	0.41	0.03	11.73***
X2: Perceived Ease of Use	−0.34	0.05	−8.99***	0.36	0.03	10.03***
X3: Information Transparency	−0.36	0.05	−9.61***	0.44	0.03	12.52***
M: Administrative Burden				−0.28	0.02	−7.88***

*p < 0.00, **p < 0.01, ***p < 0.001.

The non-parametric percentile bootstrap method was employed to assess the significance of the mediation effects. The results indicate that administrative burden partially mediates the relationship between perceived usefulness and policy compliance, with an indirect effect of 0.08 (95% *CI* = [0.06, 0.11]), supporting Hypothesis 1. Similarly, administrative burden partially mediates the relationship between perceived ease of use and policy compliance, yielding an indirect effect of 0.08 (95% *CI* = [0.06, 0.10]), supporting Hypothesis 2. Furthermore, administrative burden partially mediates the relationship between information transparency and policy compliance, with an indirect effect of 0.08 (95% *CI* = [0.05, 0.10]), supporting Hypothesis 3.

**Figure 2:**
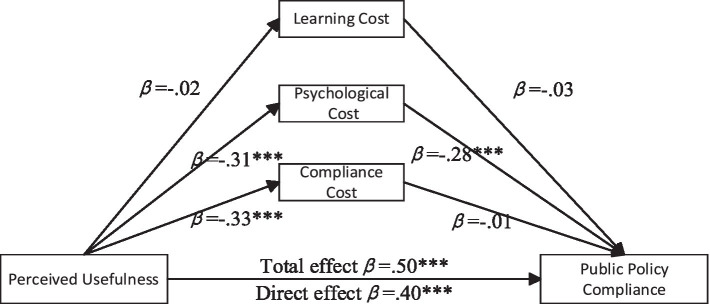
Mediation paths of perceived usefulness on public policy compliance. *p < 0.05, ***p < 0.001.

Further Exploration of the Mediating Effects of Learning Cost, Psychological Cost, and Compliance Cost on Perceived Usefulness and Policy Compliance. A simple mediation analysis was conducted using the PROCESS plugin for SPSS 26.0 (Model 4), following the methodology of Hayes (2018). Path coefficients are illustrated in Figure 2. The significance of the mediation effects was tested using non-parametric bootstrap procedures. The results show that the indirect effect of learning cost as a mediator is 0.000 (95% *CI* = [−0.003, 0.006]), the indirect effect of psychological cost is 0.088 (95% *CI* = [0.0546, 0.125]), and the indirect effect of compliance cost is 0.002 (95% *CI* = [−0.028, 0.032]). Among the three, only psychological cost was found to partially mediate the relationship between perceived usefulness and public policy compliance.

**Figure 3:**
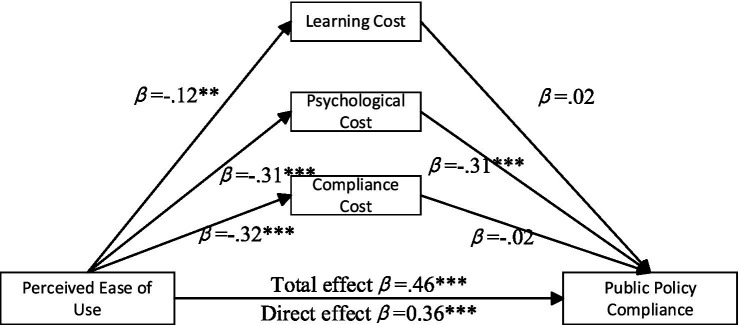
Mediation paths of perceived ease of use on public policy compliance. *p < 0.05, **p < 0.01, ***p < 0.001.

The analysis of the effects of perceived ease of use on public policy compliance highlights the mediating roles of learning cost, psychological cost, and compliance cost. As illustrated in Figure 3, the non-parametric bootstrap procedures were employed to assess the significance of these mediation effects. The results indicate that the indirect effect of learning cost is 0.003 (95% *CI* = [−0.013, 0.058]). The indirect effect of psychological cost is 0.097 (95% *CI* = [0.062, 0.136]). In contrast, the indirect effect of compliance cost is 0.007 (95% *CI* = [−0.023, 0.038]). Among the mediators, only psychological cost partially mediates the relationship between perceived ease of use and public policy compliance.

**Figure 4:**
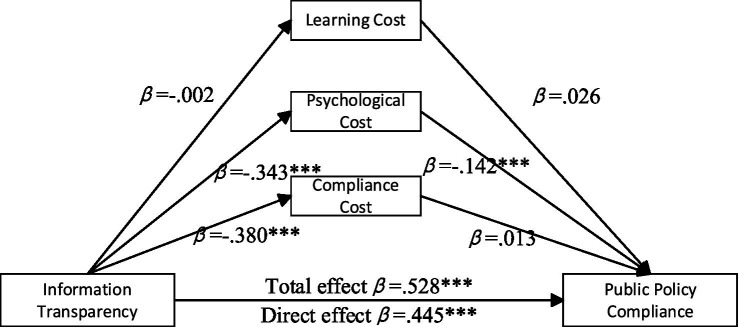
Mediation paths of information transparency on public policy compliance. *p < 0.05, ***p < 0.001.

The analysis of the effects of information transparency on public policy compliance reveals the mediating roles of learning cost, psychological cost, and compliance cost, as shown in Figure 4. The results indicate that the indirect effect of learning cost is 0.000 (95% *CI* = [−0.005, 0.0053]), which is not statistically significant. The indirect effect of psychological cost is 0.082 (95% *CI* = [0.051, 0.118]), suggesting significant mediation. The indirect effect of compliance cost is −0.009 (95% *CI* = [−0.038, 0.020]), indicating that it does not significantly mediate the relationship between information transparency and public policy compliance.

#### 3.3.4 Moderating effect of digital literacy

To further investigate the moderating effect of digital literacy, we employed Model 1 of the PROCESS plugin for SPSS 26.0 (Hayes, 2018). In this model, administrative burden was specified as the independent variable, digital literacy as the moderator, and public policy compliance as the dependent variable. Age and educational attainment were controlled in all analyses. As reported in Table 5, digital literacy significantly moderated the relationship between administrative burden and public policy compliance, consistent with Hypothesis 5.

**Table 5:** Moderating effects of digital literacy.

Dependent variable	Predictor variable	*B*	*SE*	95%*CI*		*t*	*R* ^2^	*F*
Public policy compliance	Age	0.004	0.016	−0.026	0.034	0.261	0.360	71.142***
	Educational Level	−0.045	0.040	−0.124	0.033	−1.14		
	X:Administrative Burden	−0.178	0.021	−0.219	−0.138	−8.698***		
	W:Digital Literacy	0.408	0.0356	0.338	0.478	11.479***		
	X*W	0.155	0.034	0.087	0.222	4.505***		

***p < 0.001.

### 3.4 Discussion

Study 1 confirmed regardless of the specific level of digital government; higher perceived administrative burden consistently relates to lower policy adherence. Public perceptions of digital government—specifically, perceived usefulness, perceived ease of use, and perceived information transparency—significantly enhance public policy compliance, with administrative burden functioning as a key mediator. These findings provide empirical support for Hypotheses 1–3. Further examination revealed that learning cost and compliance cost did not exert a notable mediating effect; instead, psychological cost emerged as a significant mediator. This result suggests that, during public health emergencies, the psychological ramifications of policy directives may critically shape compliance behaviors. Building upon these insights, Study 2 adopted a survey experiment that manipulated violation costs to investigate how such costs moderate the association between digital government perceptions and public policy compliance.

## 4 Study 2: conditional process model: the influence of violation costs on public policy compliance

### 4.1 Participants

A power analysis using G*Power 3.1 software indicated that, for a medium effect size (*f* = 0.25), with a significance level of *α* = 0.05, a 3 × 2 between-subjects ANOVA would require a minimum of 251 participants to achieve 95% statistical power (1 - *β*). Consequently, an online behavioral experiment was conducted via the Credamo platform. To minimize potential confounds associated with varying levels of digital literacy in administrative contexts, the sample was restricted to individuals using desktop computers. Ultimately, 312 participants were successfully recruited for the study.

### 4.2 Research design

Study 2 adopted a 3 × 2 between-subjects design to examine how digital government and violation costs jointly affect public policy compliance. Following Davis’s (1989) framework, three levels of digital government—high, medium, and low—were operationalized based on the usability and ease-of-use attributes of respective digital tools.

**Figure 5:**
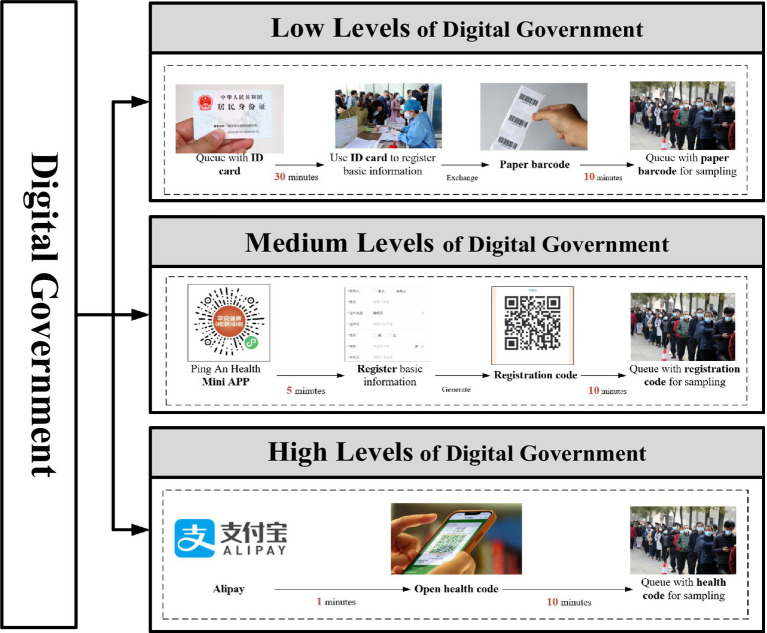
Digital government operational methods.

**Figure 6:**
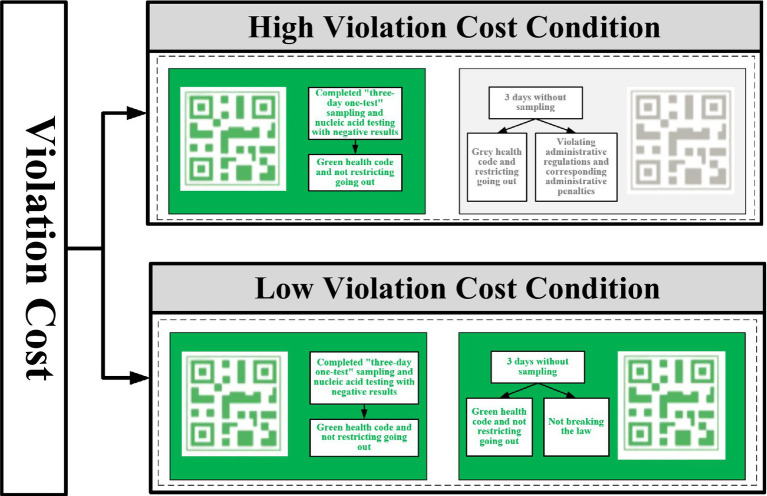
Violation cost operational methods.

The specific manipulations were structured around each tool’s time requirements for completing a health-related sampling process (see Figure 5). Under the low-digital government condition, participants used paper barcodes (40 min); under the medium-digital government condition, they engaged with a “Ping An Health” mini-program (15 min); and under the high-digital condition, they used a “Health Code” app (11 min). Violation costs were systematically varied in accordance with Hough et al. (2010). In the high-violation-cost condition, participants were informed that noncompliance would trigger a gray code, thereby restricting mobility and incurring administrative penalties. In the low-violation-cost condition, participants were instead told they would receive a green code, allowing unrestricted travel without penalties (see Figure 6). Administrative burden was assessed using the same questionnaire as in Study 1, adapted from Döring and Madsen (2022) and Madsen and Mikkelsen (2022). This scale captures core dimensions of learning cost, psychological cost, and compliance cost, thereby providing a comprehensive measure of the burdens individuals face when navigating government-imposed procedures. Perceived violation cost was measured by one question, “Imagine that you did not complete the ‘Three-Day Test’ as required and were discovered by the relevant authorities. How likely do you think you would be given an administrative penalty?” The dependent variable—policy compliance behavior—was measured as participants’ likelihood of adhering to the designated public policy under these experimentally manipulated conditions.

### 4.3 Procedure

In a 3 (Digital Government: High vs. Medium vs. Low) × 2 (Violation Costs: High vs. Low) factorial design, participants were randomly assigned to one of six experimental conditions. After excluding 30 individuals who did not pass the screening questions, the final sample included 312 valid participants, with 52 in each condition.

**Figure 7:**
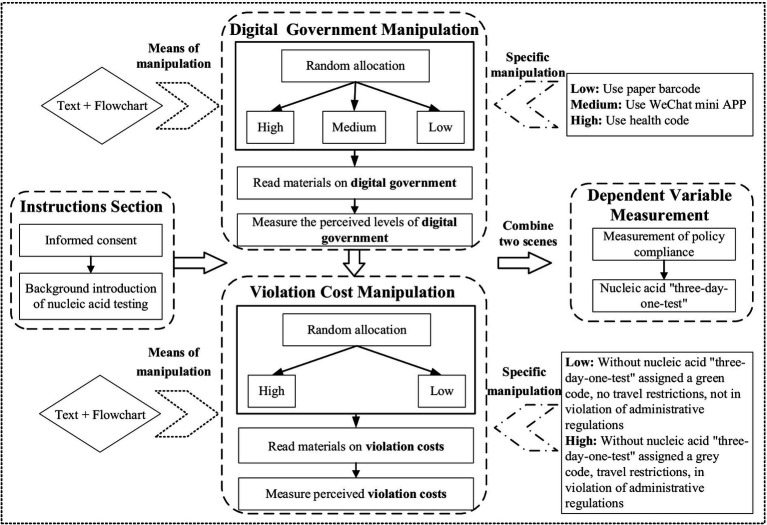
Experimental procedure flowchart.

Participants were first presented with textual explanations and flowcharts illustrating the tools used for nucleic acid sampling. They then answered the question, *“Based on the nucleic acid testing process presented above, how do you evaluate the level of digitalization for nucleic acid sampling in this context?”* to verify the effectiveness of the digital government manipulation. Next, they completed the Administrative Burden Scale. Participants were subsequently given detailed scenarios describing the potential consequences of non-compliance with the “Three-Day Test,” accompanied by further textual explanations and flowcharts to ensure clarity. They were then asked, *“If you did not complete the nucleic acid ‘Three-Day Test’ as required, would your daily life be restricted?”* to verify the effectiveness of the violation costs manipulation. To measure perceived violation cost more directly, they also responded to the question, *“Imagine that you did not complete the ‘Three-Day Test’ as required and were discovered by the relevant authorities. How likely do you think you would be given an administrative penalty?”* Finally, participants were presented with a comprehensive description that integrated both the digital government levels and violation cost conditions and were asked to report their inclination to comply with the “Three-Day Test” policy. They responded to the question *“In the context of the ongoing nucleic acid sampling environment described above, how likely are you to comply with the ‘Three-Day Test’ policy for routine epidemic prevention?”* on a 5-point scale (1 = not at all likely, 5 = very likely) (Figure 7).

### 4.4 Results and discussion

#### 4.4.1 Testing the effectiveness of the manipulations for digital government and violation costs

The manipulation check results indicated significant differences in participants’ perceptions across the low, medium, and high levels of digital government (*F* (2, 309) = 265.833, *p* < 0.01). Similarly, participants’ perceptions of violation costs differed significantly between the low and high conditions (*F* (1, 310) = 543.441, *p* < 0.01), which confirm the effectiveness of the manipulations for both digital government and violation costs.

#### 4.4.2 The impact of digital government and violation costs on policy compliance

**Figure 8:**
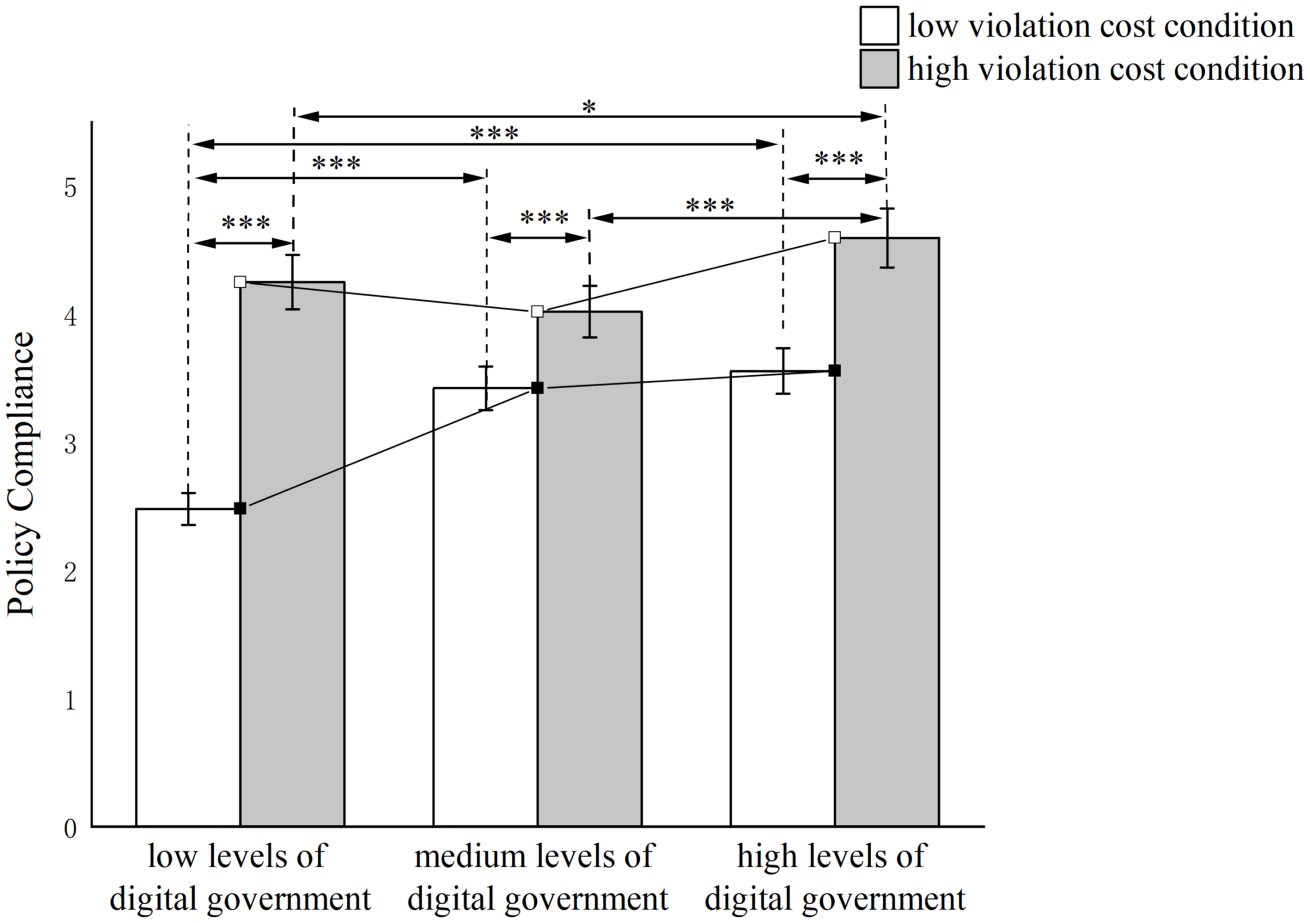
Moderating effect of violation costs.

A 3 × 2 between-subjects ANOVA revealed a significant main effect of digital government on compliance with the “Three-Day Test” policy (*F* (2, 306) = 16.259, *p* < 0.001, $\eta_{p}^{2}$ = 0.096), indicating higher policy compliance under high levels of digital government. The main effect of violation costs was also significant (*F* (1, 307) = 124.022, *p* < 0.01, $\eta_{p}^{2}$ = 0.288), with greater policy compliance observed under high violation cost conditions. The interaction effect between digital government levels and violation costs was significant (*F* (2, 306) = 11.270, *p* < 0.001, $\eta_{p}^{2}$ = 0.069). A simple effects analysis (Figure 8) indicated significant differences in policy compliance across the low, medium, and high levels of digital government under low violation cost conditions (*F* (2, 306) = 22.113, *p* < 0.001). Similarly, under high violation cost conditions, significant differences in compliance were observed across the three levels of digital government (*F* (2, 306) = 5.416, *p* < 0.01). These results suggest that Hypothesis 5b is not supported.

#### 4.4.3 An integrative model of how administrative burden and violation costs affect policy compliance across different levels of digital government

To investigate the joint impact of digital government (DG), administrative burden (Adbur), and violation costs (VC) on public policy compliance (PPC), we employed SPSS 26.0 with the PROCESS (Model 14), constructing a moderated mediation framework. As reported in Table 6, indicating that the effect of administrative burden on compliance differs by the level of violation costs.

**Table 6:** Hierarchical regression results (*N* = 312).

Variables	Administrative burden (DV: M)					Policy compliance (DV: Y)				
	*B*	*SE*	*t*	95% CI Lower	95% CI Upper	*B*	*SE*	*t*	95% CI Lower	95% CI Upper
Intercept	0.33	0.12	2.88***	0.11	0.56	3.14	0.13	25.12***	2.89	3.39
DG (X)	−0.11	0.04	−3.15***	−0.18	−0.04	0.19	0.04	5.06***	0.12	0.27
Adbur (M)						0.05	0.06	0.81	−0.07	0.17
VC(W)						0.39	0.03	12.79***	0.33	0.44
Adbur × VC (Int_1)						−0.08	0.04	−2.08***	−0.15	−0.00
*R^2^*	0.03					0.40				
*F (df)*	9.90 ***(1,310)					51.79*** (4,307)				

***p < 0.001.

The moderated mediation analysis reveals that DG’s direct effect on PPC is 0.1927 (*SE* = 0.0381, *t* = 5.0633, *p* < 0.001, 95% *CI* [0.1178, 0.2676]). Examining the conditional indirect effects of DG on PPC through Adbur at three representative values of VC shows that when VC is low (−1.8109), the effect is −0.0207 (95% *CI* [−0.0476, 0.0033]); when VC is moderate (−0.3109), the effect is −0.0080 (95% *CI* [−0.0250, 0.0067]); and when VC is high (2.1891), the effect is 0.0130 (95% *CI* [−0.0038, 0.0286]). The index of moderated mediation is 0.0084 (95% *CI* [0.0004, 0.0171]), suggesting a marginally significant moderated mediation effect whereby the indirect path from DG to PPC through Adbur shifts from negative to positive as violation costs increase, but remains modest in magnitude.

## 5 Conclusions and policy implications

This study explores the internal mechanisms and boundary conditions through which public perceptions of digital government influence policy compliance during a public health emergency. Across two progressive studies, the findings demonstrate that perceived usefulness, perceived ease of use, and information transparency significantly enhance public policy compliance, with administrative burden serving as a critical mediator. Further analysis reveals that digital literacy moderates the impact of administrative burden on compliance. Individuals with high digital literacy manage administrative burdens more effectively, reducing their negative impact on compliance. In contrast, those with low digital literacy struggle to cope with these burdens, which exacerbates their adverse effect on policy compliance. In addition, our analyses reveal that while digital government interventions can redistribute administrative burdens, only psychological cost significantly mediates the relationship between digital government perceptions and policy compliance. This finding indicates the pivotal role of psychological responses—particularly in contexts characterized by public health emergencies.

Study 2 further demonstrates that violation cost plays a pivotal role in shaping public policy compliance by interacting with digital government perceptions. In particular, higher violation cost serves to mitigate the negative impact of administrative burden on compliance, which aligns with the principles of loss aversion. The moderated mediation analysis indicates a robust direct effect of digital government on policy compliance, while the indirect effect via administrative burden shifts from negative under low violation costs to positive under high violation costs, although this shift is modest. These results suggest that the effectiveness of digital government in fostering policy compliance is contingent upon the level of enforcement costs, thereby underscoring the need for targeted enforcement strategies in digital governance initiatives.

### 5.1 Evolution of a public-friendly digital government: acceptance, compliance, and identification

Digital technologies are redefining governmental mechanisms and bureaucratic practices, thereby transforming government–citizen interactions. Consistent with the Technology Acceptance Model (TAM), this study confirms that public perceptions of usability and ease of use are key determinants in the adoption and effective use of digital government systems. Li et al. (2023) introduce the concept of public-friendly information transparency, offering a framework for making government information more accessible. By integrating the dimension of information transparency, we advocate for the development of a public-friendly digital government framework that prioritizes transparency, reliability, and accessibility.

### 5.2 A feasible path to building a public-friendly digital government: promoting scenario-matched digital government development

Developing a digital government is a comprehensive, systematic project involving data flow integration, network security, and coordinated services, while also highlighting the importance of public experience in governance efficiency (Zeng and Chen, 2024). From a practical perspective, our research identifies critical factors that facilitate public policy compliance via digital government initiatives. First, emphasizing information transparency and improving digital interface usability are essential for reducing learning, psychological, and compliance costs. Second, it is imperative to design governance scenarios tailored to diverse demographic groups—especially vulnerable populations such as the elderly, teenagers, and people with disabilities—to enhance overall administrative efficiency. Third, a balanced approach to digitization in policy implementation is necessary to maintain institutional legitimacy while reducing administrative burdens.

### 5.3 Limitations and further research

The present study demonstrates that public perceptions of digital government—specifically, its usefulness, ease of use, and information transparency—play a central role in fostering public policy compliance by alleviating learning, psychological, and compliance costs. Moreover, digital literacy is critical in enabling citizens to navigate administrative processes, thereby mitigating the adverse effects of administrative burden. Our findings indicate that under conditions of low violation costs, the advantages of well-designed digital government systems are particularly pronounced, resulting in enhanced compliance and more effective governance.

Nonetheless, several limitations warrant further investigation. First, although our research was conducted in the context of a public health emergency, the unique socio-political environment in China is characterized not only by regulatory or incentive-based mechanisms but also by a public containment policy. This raises important questions regarding how the duration of digital government application on public policy compliance. In particular, future studies should explore whether prolonged digital governance may lead to a heightened perception of punishment probability—consistent with Becker’s economic approach (Becker, 1968)—when compared with traditional paper-based control models. Second, while our results indicate that digital government is effective in updating epidemic information, the subsequent policy changes suggest that the level of transparency may be insufficient or even perceived as biased. This “blind spot” in transparency calls for a deeper investigation into how information quality and credibility affect public trust and compliance (Snijkers, 2005; Levesque et al., 2024). Third, our analysis of psychological costs suggests that sociocultural factors may also significantly influence compliance behavior. Beyond concerns over restricted movements, compliance might be driven by social pressures such as fear of stigmatization, guilt avoidance, and apprehensions related to punitive legal measures (Ball et al., 2023; Zou, 2023). Insights from Friedman’s work (Friedman, 2016) may provide a useful perspective on how legal frameworks and technological advancements interact to influence societal behavior. Future research should explore whether the identified mediating and moderating mechanisms operate similarly in routine administrative contexts and in non-emergency policy settings, such as urban management, social welfare, and environmental regulation (Larsson, 2021; Levesque et al., 2024; Zou, 2023).

## Data Availability

The raw data supporting the conclusions of this article will be made available by the authors, without undue reservation.
